# Study of Pathogenicity Test, Antifungal Activity, and Secondary Metabolites of *Bacillus* spp. from Lake Bogoria as Biocontrol of *Rhizoctonia solani* Kühn in *Phaseolu*s *vulgaris* L.

**DOI:** 10.1155/2024/6620490

**Published:** 2024-06-15

**Authors:** Tofick Barasa Wekesa, Vitalis Wafula Wekesa, Justus Mong'are Onguso, Ndinda Kavesu, Patrick Wafula Okanya

**Affiliations:** ^1^Jomo Kenyatta University of Agriculture and Technology, Institute for Biotechnology Research, P.O. Box 62000-00200, Nairobi, Kenya; ^2^Bioline Agrosciences Africa Limited Production, P.O. Box 1927-20117, Naivasha, Kenya; ^3^The Technical University of Kenya, Department of Biochemistry and Biotechnology, P.O. Box 52428-00200, Nairobi, Kenya

## Abstract

The common bean (*Phaseolus vulgaris* L.) is a yearly herbaceous plant grown for its edible dry seeds. Despite that, pests and diseases have contributed to the decline of common bean production in Kenya. Therefore, the study aimed to identify bacteria from Lake Bogoria, assess the pathogenicity of *Rhizoctonia solani* Kühn, screen for effective antifungal agents, and determine secondary metabolites for the biocontrol of *R. solani.* A total of 49 bacteria were isolated, of which 10 isolates had varied mycelial inhibition rates of *R. solani* in the co-culture technique. The efficacy of volatile compounds of the three selected bacterial strains had varied mycelial growth and percent reduction against *R. solani*. The pathogenicity assay showed varied plant parameters and biomass of *R. solani* on common bean plantlets. The molecular characterization based on 16 S ribosomal RNA confirmed the selected bacterial strains' identity with a diversity similar to the *Bacillus* genus. Gas chromatography-mass spectrometry analysis of secondary metabolites showed different antimicrobial compounds produced by *Bacillus subtilis* strain TW21. In conclusion, Lake Bogoria harbors useful microbes as biocontrol agents against plant pathogens. The current study discovers the potential biocontrol bacteria isolates from Lake Bogoria as alternative bioagents against *R. solani*. Therefore, the isolate *Bacillus subtilis* strain TW21 can be assessed further for toxicological and ecotoxicological studies and registered by the Pest Control Products Board (PCPB), Kenya, as a biocontrol product against common diseases affecting common beans' production.

## 1. Introduction

The common bean (*Phaseolus vulgaris* L.) is a herbaceous plant grown for its edible dry seeds [1]. It is categorized as dry beans, snap beans, and shell beans, with its leaves used as vegetables and fodder for animals [2]. *Phaseolus vulgaris* L is a member of the legumes family Fabaceae with a mutualistic connection with the nitrogen-fixing bacteria Rhizobium [1]. Kenya produces about 500,000 metric tons (Mt) of common beans annually Duku et al. [2]. Despite that, pests and diseases have contributed to the decline of common beans' production. Pests such as cutworms, bean flies, red spider mites, aphids, pod borers, whiteflies, and thrips are among the major factors affecting common beans' production [3]. In addition, diseases such as black root rot, damping-off diseases, bean rust, *Fusarium* wilt, and *Rhizoctonia* root rot contribute to approximately >70% loss of common bean production [4, 5]. Biotic factors such as soil infertility and environmental stress also contribute to common beans' low productivity [5]. According to Jabnoun-Khiareddine [4], a decline of >13.8% of common beans' production in 2014 was attributed to root and stem rot disease caused by *Rhizoctonia* fungi. Most Kenyan farmers for over a century have relied on synthetic fungicides as the best alternative in managing plant diseases [6]. These have been compromised, due to the ineffectiveness of fungicides and plant pathogens' adaptation mechanisms [7]. In addition, the effect of synthetic products in terms of environmental pollution leaves harmful residues on the plant, hence, expand resilient pathogen strains' repertoire. This has initiated the need for other environmentally friendly options in managing plant diseases [8]. Therefore, the implementation of integrated pest managment (IPM) in the use of biological control as a substitute management strategy has been encouraged [5, 9–12]. In Kenya, various products are registered by the Pest Control Products Board (PCPB) to be used as biopesticides. For instance, *Bacillus thuringiensis* var: *aizawai sero H7* subtype is used to control thrips and whiteflies on French beans, *Bacillus subtilis BS-01* is used to control powdery mildew on roses and rice blast in rice, and *Bacillus amyloliquefaciens strain* QST 713 is used to control coffee leaf rust, black rot, and *Alternaria* leaf spot in cabbages. In addition, Wekesa et al. [13] reported *Bacillus* spp. from Lake Magadi, Kenya, to control *Rhizoctonia solani* and also the use of *Bacillus velezensis* from Lake Bogoria to control *Fusarium solani* in common beans [14].

The antagonism of biological agents against phytopathogenic fungi are mainly through the exudation of active metabolites [15], competition for nutrients [16], induced systemic resistance [17], production of cell wall degrading enzymes [12], and production of microbial volatile compounds (mVOCs) [18, 19].

Lake Bogoria is characterized as a thermal lake due to its hot springs (>50°C). The uniqueness of the lake over the period has been an exploitation center for industrial and agricultural benefit. For instance, endophytic bacteria against *Fusarium solani* [20] and *Bacillus velezensis* against *Fusarium solani* [14] have been characterized from Lake Bogoria. In addition, different strains from soda lakes have been reported with the potential to manage plant diseases [21–24]. The study aimed to isolate bacteria from Lake Bogoria, assess the pathogenicity of *R. solani* in common bean plants, and screen effective antifungal agents to control *R. solani*. The effective strains were assayed for the efficacy of producing volatile compounds and for molecular characterization. The secondary metabolites produced by the selected antagonistic bacterial strains were identified using the gas chromatography/mass spectrometry (GC-MS) technique.

## 2. Materials and Methods

### 2.1. Sampling Site and Sample Collection

The samples were collected at five different sites of Lake Bogoria, where 100 ml of water and 50 g of soil were collected in triplicate and separately packed in sterile tubes. The physiochemical parameters such as temperature, pH, salt, total dissolved solids (TDS), and conductivity were recorded. The samples were labelled and stored at 4°C at the Institute for Biotechnology Research Laboratory at Jomo Kenyatta University of Agriculture and Technology for isolation, identification, and screening.

### 2.2. Isolation of Bacteria from Lake Bogoria

The isolation of bacteria from the soil and water was done according to Wekesa et al. [14]. In brief, 9 millilitres of sterile physiological saline (0.85% NaCl) were added to a sterile test tube in which 1 gram of soil was added and then homogenized. After that, the resultant suspensions were vortexed at 150 rpm for one minute. The suspensions were serial diluted from a concentration ratio of 1 : 9 to a dilution of 10^−4^. On modified nutrient agar—Himedia, [10.0 g/L peptone, 10.0 g/L beef extract, 5.0 g/L NaCl, and 12.0 g/L agar, 0.01 mg/L cycloheximide, 0.35% (w/v) NaCl] an aliquot of 30 *µ*l was cultured from dilutions of 10^−3^ and 10^−4^, according to Hartman [25]. The plates were incubated at 35°C for 48 hours, and based on colony morphology, distinct colonies were separated and cultured on an isolation medium to obtain pure cultures. The pure bacteria cultures were characterized using standard microbiological techniques. Cell morphology was done using the Gram stain technique as described by Tripathi and Sapra [26] using a light microscope (MD827S30L).

### 2.3. Pathogenicity Assay

#### 2.3.1. Culturing and Inoculum Preparation

The fungal strain used in this study was *Rhizoctonia solani* Kühn (ATCC 66150), obtained from Bioline Agrosciences African Limited, Naivasha, Kenya. The fungal strain was subcultured on potato dextrose agar-Himedia [200.0g/L Potatoes, 20.0 g/L Dextrose and 15.0 g/L Agar] (PDA) medium and incubated at 30°C for 7 days. The *R. solani* inoculum preparation was done as described by Jabnoun-Khiareddine [4]. In brief, the mycelium of *R. solani* was scrapped off from the 7 days old culture from PDA plate and mixed with sterilized distilled water (SDW). It was then stored at 4°C for further experiment.

#### 2.3.2. *In Vitro* Assay of Pathogenicity Test

*In vitro* pathogenicity assay was done as described by Asaka and Shoda [27]. In brief, the forest soil was autoclaved for 60 mins at 121°C thrice at 12 hours intervals. Approximately 180 g of sterilized soil was packaged in sterilized plastic containers of maximum water-holding capacity. Five days before sowing the common bean seeds, the soil was inoculated with *R. solani* at 5 : 1 (5 mL of *R. solani* inoculum: 1 pot), whereby SDW was used as a negative control. The plant assay was done as reported by Rocha et al. [28], where seeds were surface sterilized by first socking with 70% (v/v) of ethanol for 5 minutes and then 0.5% of sodium hypochlorite (NaOCl) for one minute. Two seeds were planted in each pot after being washed three times with SDW to remove NaOCl and air dried. They were placed in the growth chamber at 8 hours a day and 16 hours a night at 28°C with 80% relative humidity. The severity of *R. solani* symptoms was evaluated two weeks after the initial inoculum using a scale from 1 to 7, as described by Godoy et al. [29]. In addition, plant parameters such as shoot length, root length, plant height, and biomass were measured and recorded.

### 2.4. Antibiosis Assay of Bacterial Isolates against *R. solani*

The antibiosis assay of bacterial isolates was assessed against a test pathogen as described by Aydi et al. [10]. The PDA medium was used to culture fungus and bacteria by placing an active growing plug at the end of the petri dish and streaking bacteria (10^8^ CFU/mL) perpendicularly across the plate. In contrast, the pathogen alone was used as a control. The plates were incubated at 30°C for 14 days, and the percentage inhibition rate (%I.R) was calculated using the formula as described by Aydi et al. [10]. The mycelium length was measured using a ruler in centimeter (cm).

(1) $$\begin{array}{c} \%I.R=\frac{C2-C1}{C2}\times 100, \end{array}$$

where *I*.*R* is the inhibition rate, *C*2 is the Colony diameter of the pathogen in control, and *C*1 is the colony diameter of the pathogen cocultured with bacteria.

### 2.5. Effect of Volatile Compounds by Selected Bacteria against *R. solani*

To determine the efficacy of the selected bacteria to produce VOCs, paired disc technique was used as described by Nigris et al. [30] and Nikitin et al. [31]. The selected bacteria were uniformly spread onto Nutrient Agar (N.A)—Himedia medium and a plug (6 mm) of active growing *R. solani* were punched and placed at the center of the PDA medium plate. The two plates were sandwiched, whereby PDA medium plate with fungi were placed at the bottom and N.A medium plate with bacteria placed on top. They were sealed and incubated at 28°C for 7 days. The percentage suppression of the pathogen was determined as described by Garbelotto et al. [32].

### 2.6. Molecular Characterization of the Selected Bacterial Isolates

The antagonistic bacterial isolates were identified by amplifying and sequencing the 16 S ribosomal RNA (rRNA) gene. Genomic DNA was extracted using a bacterial DNA isolation kit (Norgen Biotek Corp. Thorold, ON, Canada) according to the manufacturer's instructions. The genomic DNA was then quantified using Qubit (Qubit 4 Fluorometric quantification, Q33238), and its final concentration was adjusted to 100 ng of DNA/*µ*l.

The universal bacterial primers used for the amplification were 27 F (5′-AGAGTTTGATCCTGGCTCAG. 3′) and 1492. R (5′-CGGCTACCTTGTTACGACTT-3′). The Peqlab Primus 96 thermocycler (PEQLAB, Erlangen, Germany) was used for PCR amplification. The PCR solution was prepared by mixing 20.0 *μ*l of master mix, 18.2 *μ*l of PCR water, (10.0 ppm) of 0.4 *μ*l of the 27-F primer, (10 ppm) of 0.4 *μ*l of the 1492-R primer, and 1.0 *μ*l of template DNA (10.0 ng/mL) in a final volume of 40.0 *μ*l. The reaction mixtures were subjected to temperature cycling profiles as described by Wekesa et al. [14]. The PCR products were confirmed by running a 1.5% agarose gel that was viewed under a UV gel documentation system. The PCR products were then purified using QIAquick PCR amplification kit (Qiagen, Hilden, Germany), and sent to Macrogen (South Korea) for sequencing.

### 2.7. Sequence Analysis and Phylogenetic Tree Preparation

ChromasPro V2.0 software was used to edit the Sanger sequencing data of the bacterial isolates, and a nucleotide BLAST search performed on the National Center for Biotechnology Information (NCBI) database. The sequences were selected based on their similarity to reference genome sequences retrieved from NCBI, and multiple alignments were performed using the MAFT plug-in for Geneious PrimeV2023.1.2 software. Maximum likelihood analysis was used to construct the phylogenetic tree for datasets in GeneiousPrime 2023.1.2 RAxML plug-in by looking for the highest-scoring ML tree among 1000 iterations under the GTR-gamma model with rapid bootstrapping. The sequences were submitted to the GenBank with accession PP301463, PP301464, and PP301465.

### 2.8. Extraction of Secondary Metabolites from Effective *Bacillus* spp

Following the method outlined by Yehia et al. [33], the crude metabolites were extracted, whereby *Bacillus subtilis* strain TW21 was cultured in nutrient broth [10.0 g/L Peptone, 10.0 g/L Beef extract, 5.0 g/L NaCl] (N.B)—Himedia, at 28°C for 3 days. The supernatant was extracted by centrifuging the sample at 8000 rpm for 30 minutes at 28°C. The extracted supernatant was stirred for 8 hours at 28°C and 100 rpm in an orbital shaker, and then acidified to a pH of 2.0 using concentrated HCl. The antifungal chemicals were extracted by adding an equivalent amount of ethyl acetate to the culture broth and shaken for 2 hours at 200 rpm in an orbital shaker. The culture broth was extracted twice using ethyl acetate and evaporated at 60°C and 80 rpm to obtain a concentrated antifungal crude extract. The crude extract was dissolved in 1 ml of methanol: chloroform mixture (1 : 1) and analyzed on GC/MS machine.

### 2.9. Characterization of Secondary Metabolites by GC-MS

The Shimadzu QP-2010 GC-MS (Kyoto, Japan) equipment with a capillary column (inner diameter 0.25 mm and length 30 m) was used to analyze the crude extract of *Bacillus subtilis* strain TW21. The GC oven was preheated to 100°C for 2 minutes, then set to rise to 280°C at a rate of 10°C per minute, and finally held at 280°C for 13 minutes. A volume of 2 mL of crude extract of *Bacillus subtilis* strain TW21 was injected into a split ratio of 1 : 0.25 yielding a total volume of 0.5 mL of pure extract. Both the injector and detector ports were heated to a comfortable 200°C. The energy level for electron ionization in a GC-MS was 150 eV. The mass range examined was 20–500 amu at a scan duration of 70 ms. Mass spectrometry was used to analyze the gas chromatogram peaks. The active ingredients were determined using retention indices and mass spectra compared to the library of mass spectra maintained by the National Institute of Standards and Technology.

### 2.10. Data Analysis

The pathogenicity and coculture data were analyzed using statistical analysis software (SAS) version 8.0 software, while all graphs were analyzed using GraphPad-Prism version 6.0.

## 3. Results

### 3.1. Isolation of Bacteria

**Figure 1:**
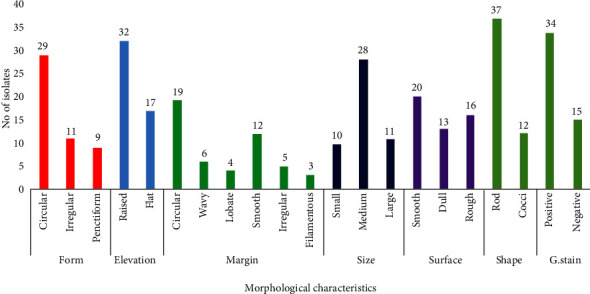
Morphological characteristics of the isolated bacterial colonies.

A total of 49 bacterial isolates were obtained from 5 sampling points of Lake Bogoria. The isolates were characterized by colony and cell morphology (Figure 1). The shape of colonies with round had 29 isolates, irregular (11), and penctiform (9) isolates. In addition, the elevation of colonies recorded 32 isolates with raised elevation, while 17 were flat. The colony margin varied from entire (19), wavy (6), lobate (4), irregular (5), and filamentous (3). The colony size in millimetre showed that most of the isolates had medium size (28), followed by large (11), and small (10). The colony's surface varied from smooth to rough, with the largest number having a smooth surface. There were more Gram-positive (34) isolates than Gram-negative (15) isolates (Figure 1).

### 3.2. Test for Pathogenicity of *Rhizoctonia solani*

**Figure 2:**
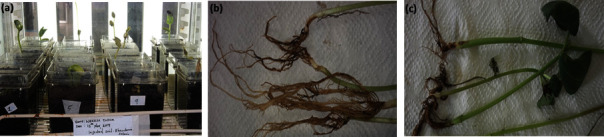
Pathogenicity test of *R. solani* on common bean plantlets: (a) growth chamber, (b) control, and (c) *R. solani*.

There was no necrotic lesion detected on the roots and stem of control treatment (Figure 2(b)), whereas the *R. solani* treatment exhibited lesions ranging from up to 30 mm in length to a large discoloured dry lesion (Figure 2(c)). In addition, the roots were long and healthy in the control treatment compared to *R. solani* treatment (Figure 2). Furthermore, the germination rate of *R. solani* was not aggressive, with the lowest seed germination rate (63.00 ± 0.58%b), compared to the control (94.00 ± 0.58%a). The fungal treatments significantly affected the plant height of common bean plantlets compared to the control. There was a significant difference (*P* < 0.05) amongst *R. solani* and control in plant parameter and biomass (Table 1).

The common bean plantlet biomass differed significantly (*P* < 0.05) upon treatments, with *R. solani* treatment having lower biomass weights than the control. From the results (Table 1), there was a significant difference between test fungal and control in plant biomass. The severity of the common bean plantlets indicated that *R. solani* had the highest number of dead plants (37.50 ± 1.20b%) compared to the control (0.00 ± 0.00a%) (Table 1).

### 3.3. Antibiosis of Bacterial Isolates against Selected Test Pathogen

**Figure 3:**
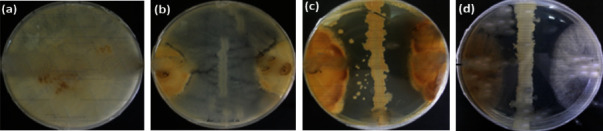
Antibiosis assay of positive Lake Bogoria bacterial isolates against *R. solani* after 14 days of incubation at 30°C ± 2.0: (a) *R. solani* only, (b) BW21 and *R. solani*, (c) BW07 and *R. solani*, and (d) BW20 and *R. solani*.

A clear zone of inhibition was observed in ten out of forty-nine isolated bacteria against *R. solani*, indicating the antifungal ability of the bacterial isolates when in direct contact (Table 2). However, the inhibition rate differed depending on the isolate interacting with *R. solani*. Isolates mainly drove the variation, as post hoc analysis revealed variation among the isolates (Figure 3). In contrast, much higher variation was observed amongst the isolates (*F*_10_ = 124.72, *P*=0.05, Table 2), and ANOVA analysis showed that BW21 was the most inhibitory strain, followed by BW07 and BW20, which showed modest levels of inhibition (*P* < 0.05). The isolate BW35 had the smallest inhibition zones against *R. solani* (*P* < 0.05). The results also suggest that Lake Bogoria isolates inhibit the mycelium growth of *R. solani in vitro*, which varied amongst the isolates (Table 2). The bacterial isolates with >30.00% inhibition rate were selected for further analysis.

### 3.4. Effect of Volatile Compounds by Selected Bacteria against *R. solani*

**Figure 4:**
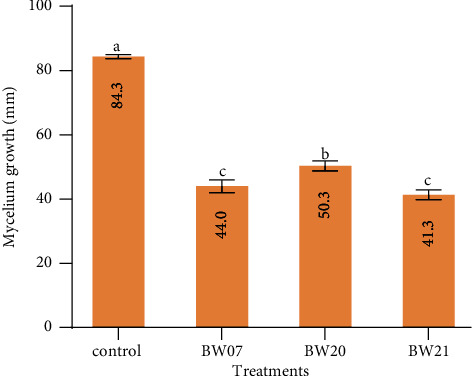
Efficacy of volatile compounds from selected bacterial isolates against the growth of *R. solani* under *in vitro* conditions. According to Fisher's HSD test (*P* < 0.05), different superscript letters (a, b, and c) indicate significantly different means within each parameter. The error bars indicate a standard error (SE). The BW21: *Bacillus subtilis* strain TW21, BW07: *Bacillus subtilis* strain TW7, and BW20: *Bacillus subtilis* strain TW20. The data are an average of three replicates.

The production of VOCs of the selected bacterial isolates showed varied significant differences in the length of the mycelium growth across the treatments. However, the control had maximum mycelium growth of 84.00 mm with the lowest percentage reduction of 0.00% (Figure 4). Isolate BW021 had the lowest mycelium growth (41.00 mm) and the highest percentage reduction of 51.19%. Isolate BW07, on the other hand, recorded the same mycelium growth (Figure 4). Isolate BW07 recorded slightly lower mycelium growth compared to BW20; however, it had a higher percentage reduction compared to isolates BW07 and BW21.

### 3.5. Molecular Characterization of Selected Antagonistic Isolates

**Figure 5:**
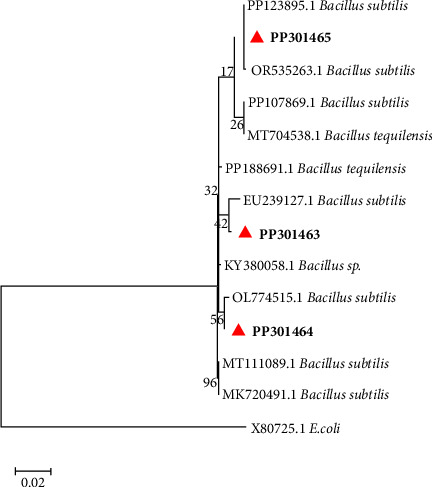
The evolutionary relationship of the selected bacterial isolates using maximum likelihood-based in the GTR-gamma model. A total of ten nucleotide sequences were selected based on their similarity to reference genome sequences retrieved from NCBI, and multiple alignments were performed using the MAFT plug-in. Maximum likelihood analysis was used to construct the tree using RAxML plug-in with rapid bootstrapping of 1000 iterations under the GTR-gamma model. A scale bar of 0.02 was used.

The molecular sequencing performed on three selected bacterial isolates showed the highest similarity identity >99.77% with the *Bacillus* genus (Figure 5). Among the identified strains are *Bacillus* sp. and *Bacillus velezensis* (Figure 5). The phylogenetic analysis of the isolates classified into two groups. The first group comprises B7 and B21, affiliated with *Bacillus* sp. and *Bacillus subtilis*. The second group comprises B20, affiliated with *Bacillus velezensis* strain QH03–23, with a similarity index of 99.89% (Table 3).

### 3.6. *Bacillus subtilis* Strain TW21 Secondary Metabolite Characterization by Gas Chromatography/Mass Spectrometry (GC-MS)

**Figure 6:**
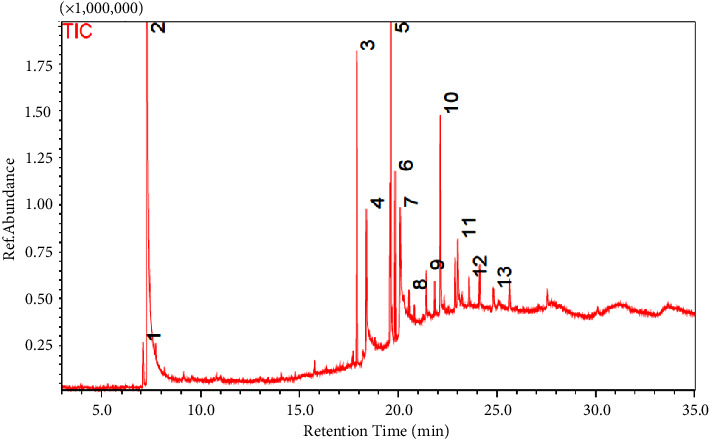
Gas chromatography/mass spectrometry chromatograms of secondary metabolites detected by solvent-free solid injection from *Bacillus subtilis* strain TW21. TIC: total ion chromatogram/current and 1-13: different peaks.

The GC/MS results identified various antimicrobial compounds produced by *Bacillus subtilis* APU-T03 (Table 4; Figure 6). The crude metabolites of the *Bacillus subtilis* APU-T03 were analyzed by GC/MS to determine the production of antimicrobial compounds. The compounds identified were confirmed using the NIST library. The most detected antifungal compounds were 3-heptanone, 5-ethyl-4-, pyrrolo [1,2-a] pyrazine-1,4- dione, hexahydro, 9-octadecen-1-ol, (Z)-, cyclononasiloxane, octadecamethyl-, and benzeneacetic acid (Table 4; Figure 6). In addition, the results revealed antimicrobial compounds produced by *Bacillus subtilis* APU-T03 such as L-5-propylthiomethylhydantoin, 1-propanol, 2,2-dimethyl-, acetate, butanoic acid, 2-methyl-, 1-hexadecanol, n-nonadecanol-1, 2,5-piperazinedione, 3,6- bis(2-methylpropyl)-, and Bis(2-ethylhexyl) phthalate (Table;4; Figure 6).

## 4. Discussion

Control of *R*. *solani* has faced many challenges since no lasting control strategies from synthetic, semisynthetic, or biological products are currently used for their control and management. These pathogens attack more than 500 legume species contributing to low-yield production among small-scale farmers [7, 45]. The results from pathogenicity showed that *R*. *solani* affected common bean plantlet germination rates, severity, plant biomass, and plant parameter. In addition, a reddish-brown lesion was observed on the plantlets' roots indicating the effect of *R. solani* on the bean plantlets. These findings agree with previous studies on the pathogenicity of *R*. *solani*, which is associated with stunting growth due to the ability of the pathogen to affect the plant's roots limiting uptake of nutrients [4, 5, 10, 46, 47].

The BLAST result based on 16 S rRNA showed taxonomic classes of the isolated *Bacillus* strains. These findings agree with Lake Bogoria's earlier investigations, which found that Firmicutes are the preponderant bacterial kingdom [23]. In addition, *Bacillus* spp. is one of the most common aerobic, eubacterial alkaliphiles in soda lakes and other natural environments [48].

Bacteria isolates obtained from Lake Bogoria showed high inhibition activity on the mycelial growth of *R. solani* in coculturing and by production of volatile compounds. The mechanism can be due to the production of various lytic enzymes involved in cell degradation during antagonism [12]. Some isolates had high inhibitory activity, while others showed limited activity, indicating the types of antifungal metabolites produced may vary [5].

Different bacteria, such as *Bacillus* spp., are well known to produce various antibiotics biocontrol agents of plant diseases. In addition, endophytes bacteria have been reported to have antibiosis effects against *R. solani* [28] using antibiosis as the most important mechanism to limit plant pathogen invasion. It also inhibited the development of plant pathogenic organisms by producing secondary metabolites [12, 49]. Different studies have been carried out in biological control, aiming to find the best solution for the control and management of *R. solani*. For instance, Belete [50] reported native *Bacillus* isolates to eradicate black root rot diseases initiated by *F. solani* in faba beans; Jabnoun-Khiareddine [4] reported the application of fungal and bacterial agents to control root rot disease in pepper. In addition, Aydi et al. [10] reported using endophytic bacteria from *Datura stramonium* to manage *Fusarium* wilt disease in tomatoes, and lastly, Mahmoudi and Naderi [11] reported the antifungal and biocontrol properties of chitinolytic bacteria in control of *Fusarium* root rot in safflower and the impact of biocontrol on *Rhizoctonia* diseases on potatoes. Lastly, Chen et al. [51] reported the effect of bichar and *Bacillus subtilis* to effectively reduce disease incidence and disease index in radish plants affected by *R. solani*.

The production of VOCs has attracted growing attention as a biocontrol mechanism since there is interaction between microorganisms and the environment. From our findings, the mycelium growth of *R. solani* was inhibited by bacterial strain TW7, TW20, and TW21. This is consistent with the previous study of bacterial VOCs inhibiting fungal and bacterial plant pathogens [52–54]. The study on *Bacillus* VOCs antagonistically against phytopathogen has been also reported by Bruisson et al. [55]. Other research has reported *Bacillus* spp. ACB-65 and *Bacillus* spp. ACB-73 produced volatile chemicals that inhibited *Phyllosticta citricarpa* by 86% [56].

In this work, secondary compounds such as pyrrolo (1,2-a) pyrazine-1,4-dione, hexahydro-, 9-octadecenol, 1-propanol, 2,2-dimethyl-acetate, butanoic acid, 2-methyl-, N, NDimethyl, 3-heptanone, 5-ethyl-4-methyl-, phenol, and benzoic acid were among the most significant antifungal chemicals found. The findings agree with Surya et al. [57], who reported the antimicrobial activity of fatty acid salts of N-N-dimethyl. The research done by Bharose and Gajera [58] also reported *B. subtilis* strain JNDKHGn-29-A to produce antifungal metabolites such as bis (2-ethylhexyl) phthalate and pyrrolo [1,2-a]pyrazine-1,4-dione. Our findings are also consistent with the findings by Wu et al. [59], who found that *Bacillus* spp. produce secondary metabolites with antifungal activity such as bacillomycin, fengycin, iturin and sufactin in control of *R. solani* in peppers. According Wu et al. [59], these compounds affect the spore germination and membrane permeability of *Fusarium*, *Rhizoctonia*, and *Alternaria*. The extraction of the lipoprotein has been reported to have a strong inhibitory effect on the growth of *R. solani* [30]. Finally, Li et al. [60] reported *Bacillus subtilis* SL-44 to produce L,D-transpeptidase and D-alanine carboxypeptidase which play an important role in the peptidoglycan cross-linking and also glutamate which are used for cell wall synthesis. In addition, the metaboic analysis of *Bacillus subtilis* SL-44 showed the presence of athyl ester, L-pyroglutamic acid, and L-alanosine which are used to synthesise drugs in control of fungal diseases. Our results also agrees with Jangir et al. [61] who found antifungal activity against *Fusarium oxysporum* from compounds in *Bacillus* sp., including N, N-dimethyl-1,2-benzene dicarboxylic acid and 9-octadecenoic acid.

## 5. Conclusion

Lake Bogoria harbors diverse microbes with different morphological characteristics. The pathogenicity test showed the ability of *R. solani* to illicit necrotic lesions and impact on plant biomass. The antifungal activity indicated the ability of the bacteria isolates to inhibit the growth of *R. solani in vitro* and capability to suppress the mycelium through production of secondary metabolites. This has been quantified through GC-MS and identified metabolite compounds with both antifungal and antibacterial activities. Therefore, further research is required to address the toxicological and ecotoxicological studies of *Bacillus subtilis* TW21 to be registered as a biocontrol product.

## Figures and Tables

**Table 1:** Comparative effects of *R. solani* on common bean plantlets observed 14 days of inoculation.

Treatments	Germination rate (%)	Plant height (cm)	Severity (%)	Root length (cm)	Root fresh weight (g)	Shoot length (cm)	Shoot fresh weight (g)	Symptom (scale 1–7)
Control	94.00 ± 0.58^a^	34.28 ± 0.10^a^	0.00 ± 0.00^a^	10.08 ± 0.45^a^	0.33 ± 0.02^a^	24.12 ± 0.45^a^	1.65 ± 0.05^a^	1.00 ± 0.00^a^
*R. solani*	63.00 ± 0.58^b^	18.28 ± 0.56^b^	37.50 ± 1.20^b^	3.80 ± 0.15^b^	0.24 ± 0.01^b^	13.08 ± 0.27^b^	0.91 ± 0.02^b^	6.00 ± 0.58^b^
*F* value	1441.50	779.71	1035.08	194.27	33.88	464.60	169.40	75.00
*P* value	0.0001	0.0001	0.0001	0.0002	0.004	0.0001	0.0002	0.001
HSD	2.27	1.57	3.33	1.32	0.08	1.46	0.17	1.60
CV	1.27	2.64	7.61	8.30	11.48	3.35	5.92	20.20

The data are average of three replicates. Results of the comparative effect of *R. solani* are shown as mean values (±SE). Following significant one-away ANOVA, subsequent Tukey's honest significant difference (HSD) test at *P* < 0.05. Means in a column followed by the same letter do not significantly differ.

**Table 2:** Antifungal activity of Lake Bogoria bacterial isolates on mycelium growth of *R. solani*.

	Isolate code	Mycelium length (cm) (mean ± SE)	% inhibition rate
Bogoria	Control	8.4 ± 0.00^a^	0.00
	BW35	7.77 ± 0.59^b^	7.54
	BW04	7.13 ± 0.15^c^	15.08
	BW23	6.83 ± 0.25^cd^	18.65
	BW39	6.63 ± 0.21^de^	21.43
	BW38	6.40 ± 0.36^def^	23.81
	BW30	6.37 ± 0.40^ef^	24.21
	BW31	6.00 ± 0.20^fg^	28.57
	BW20	5.90 ± 0.26^gh^	30.95
	BW07	5.50 ± 0.17^hi^	34.52
	BW21	5.20 ± 0.17^i^	38.10
	CV	1.71	
	*F* value	124.72	
	HSD	0.22	
	*P*>	0.05	

The percent inhibition rate (%IR) was calculated after 14 days. Mean values (±SE) in a column followed by the same letter do not significantly differ according to the Tukey HSD test (*P* < 0.05). The data are an average of three replicates.

**Table 3:** The molecular identification based on BLAST analysis of 16S rRNA genes sequencing with corresponding GenBank accession number of 3 selected bacterial strains.

Bacterial strain ID	Acc. no	GenBank closest relative (accession no.)	Per. identity (%)
TW7	PP301463	*Bacillus subtilis* strain KNUC370 (EU239127.1)	98.55
TW20	PP301465	*Bacillus subtilis* strain S1 (PP123895.1)	98.57
TW21	PP301464	*Bacillus subtilis* strain Pb2441 (OL774515.1)	99.30

**Table 4:** The retention times (RT), chromatographic relative area percentages, molecular weight, and functional activity of the secondary metabolites generated from *Bacillus subtilis* strain TW21 identified by gas chromatography/mass spectrometry solvent-free solid injection.

Peak no.	Retention time	Compound name	Area (%)	Molecular formula	Molecular weight	Functional activity	References
1	7.090	Pentanoic acid and 3-methyl-4-oxo-	2.10	C_6_H_10_O_3_	130	Antibacterial activity	[34]

2	7.290	L-5-Propylthiomethylhydantoin	55.17	C_7_H_12_N_2_O_2_S	188	Antimicrobial activity	[35]

3	17.910	3-Heptanone and 5-ethyl-4-methyl-	7.03	C_10_H_2_0O	156	Antifungal	[36]

4	18.394	1-Propanol, 2,2-dimethyl-, and acetate	4.01	C_7_H_14_O_2_	130	Antimicrobial activity	[37]

5	19.629	Butanoic acid and 2-methyl-	11.02	C_5_H_10_O_2_	102	Antimicrobial	[38]

6	19.840	1-Hexadecanol	3.92	C_16_H_34_O	242	Antimicrobial activity	[39]

7	20.105	Pyrrolo [1,2-a] pyrazine-1,4-dione and hexahydro	4.29	C_11_H_18_N_2_O_2_	210	Antifungal activity	[40]

8	20.550	9-Octadecen-1-ol and (Z)-	0.40	C_18_H_36_O	268	Antifungal and antibacterial activity	[41]

9	21.430	n-Nonadecanol-1	1.49	C_19_H_40_O	284	Antimicrobial and cytotoxic properties	[42]

10	22.139	2,5-Piperazinedione and 3,6-bis(2-methylpropyl)-	6.58	C_12_H_22_N_2_O_2_	226	Antimicrobial activity	[43]

11	23.019	Bis(2-ethylhexyl) phthalate	2.29	C_24_H_38_O_4_	390	Antimicrobial activity	[44]

12	23.591	Cyclononasiloxane and octadecamethyl-	1.00	C_18_H_54_O_9_Si_9_	666	Antifungal activity	[43]

13	24.820	Benzeneacetic acid	0.69 100.00	C_8_H_8_O_2_	136	Antifungal and antimicrobial	[41]

## Data Availability

The data used to support the findings of this study are available from the corresponding author upon request.
